# Supplementary material for: Local Adaptive Mapping of Karyotype Fitness Landscapes
Source: bioRxiv. 2025 Jun 21:2023.07.14.549079. Preprint. [Version 3] doi: 10.1101/2023.07.14.549079 (PMC12262491; doi:10.1101/2023.07.14.549079)
Supplement: Supplement 1 [file NIHPP2023.07.14.549079v3-supplement-1.pdf]

bioRxiv preprint doi: https://doi.org/10.1101/2023.07.14.549079; this version posted June 21, 2025. The copyright holder for this preprint (which was not certified by peer review) is the author/funder, who has granted bioRxiv a license to display the preprint in perpetuity. It is made available under aCC-BY 4.0 International license.

Supplementary Information

1 The ALFA-K Method: Inferring and Forecasting Karyotype Evolution

Here, we provide the full details of our ALFA-K (Adaptive Local Fitness landscape for Aneuploid Karyotypes) inference method. ALFA-K infers karyotype fitness from longitudinal count data and uses this information to forecast evolutionary trajectories. Central to our approach is the concept of fitness, which we interpret as the intrinsic net growth rate associated with each distinct karyotype. We assume that the observed karyotype counts obtained at different time points are the result of uniform sampling from the underlying population. This sampling process links the observed data to the unobserved, true relative frequencies of karyotypes within the population.

1.1 Overview of the ALFA-K Workflow

ALFA-K takes as input a longitudinal count matrix Y, whose entry y_{it} is the count of karyotype i at time t. The core workflow involves three main inference stages (items 1–3) followed by validation (4) and forecasting (5):

1. **Frequent Karyotype Fitness Estimation:** Identify frequently observed karyotypes and estimate their fitness using replicator dynamics.
2. **Single-Step Neighbor Fitness Estimation:** Extend fitness estimates to karyotypes that are one missegregation event away from frequent karyotypes.
3. **Global Landscape Inference via Kriging:** Use Gaussian process regression (Kriging) to interpolate fitness across the broader karyotype space, leveraging the estimates from frequent clones and their neighbors.
4. **Internal Consistency Check:** Employ a cross-validation procedure to assess the reliability and generalization capability of the inferred landscape.
5. **Evolutionary Forecasting:** Utilize the inferred fitness landscape within a simulation framework to predict future population dynamics.

Each stage is detailed below.

1.2 Step 1: Fitness Estimation for Frequent Karyotypes

We begin by focusing on inferring the fitness of frequently observed karyotypes. A key simplification in this step is modeling these types as effectively present from the beginning of the observation window, each with an initial frequency estimated directly from the data alongside its fitness (f_i). This approach circumvents the need to reconstruct potentially complex and underdetermined evolutionary pathways leading to these frequent states, while still accommodating clones that may have emerged later (captured by a small fitted initial abundance). Further simplifying assumptions for this step include neglecting stochastic extinction (acceptable due the inference's low sensitivity to the distinction between rare and absent states), mutations between these frequent types, density-dependent growth, and frequency-dependent selection. By making these targeted simplifications, we obtain robust, data-driven fitness estimates for the most prevalent karyotypes, which serve as a critical foundation for the subsequent steps of the pipeline.

bioRxiv preprint doi: https://doi.org/10.1101/2023.07.14.549079; this version posted June 21, 2025. The copyright holder for this preprint (which was not certified by peer review) is the author/funder, who has granted bioRxiv a license to display the preprint in perpetuity. It is made available under aCC-BY 4.0 International license.

1.2.1 Definition and Model

A karyotype i is deemed frequent if its total count across all time points satisfies $\sum_t y_{it} \geq N$, for a user-defined threshold N. Let S be the set indexing these frequent karyotypes. We model the dynamics of each frequent karyotype $i \in S$ using the continuous-time replicator equation, which describes frequency changes under selection:

$$\frac{dx_i(t)}{dt} = x_i(t)\Big[f_i - \sum_{j \in S} x_j(t)\, f_j\Big], \tag{S1}$$

where $x_i(t)$ is the frequency of karyotype i at time t relative to other frequent karyotypes in S, and f_i is its constant, intrinsic fitness parameter (net growth rate).

Interpretation (Replicator vs. Exponential Growth): The replicator equation emerges naturally from assuming independent exponential growth for each karyotype. If the absolute abundance $y_i(t)$ of karyotype i follows $\frac{dy_i}{dt} = f_i\, y_i(t)$ (leading to $y_i(t) = y_i(0)\, e^{f_i t}$), then its relative frequency $x_i(t) = \frac{y_i(t)}{Y_{\text{tot}}(t)}$, where $Y_{\text{tot}}(t) = \sum_j y_j(t)$, indeed satisfies Equation S1. Thus, inferring f_i under the replicator model is equivalent to inferring the exponential growth rate from relative frequency data.

1.2.2 Fitness Inference Procedure

We estimate the fitness vector $\mathbf{f} = \{f_i\}_{i \in S}$ in two steps:

Initial Estimation via Quadratic Programming (QP) To obtain initial fitness estimates, we linearize the replicator dynamics over short time intervals Δt. Let $x_{it} = y_{it}/N_t$, where $N_t = \sum_{j \in S} y_{jt}$ is the total count of frequent karyotypes at time t. The discrete frequency change is $\Delta x_{i,t} = (x_{i,t+1} - x_{i,t})/\Delta t$. In vector form, Equation S1 can be written as $\frac{d\mathbf{x}_t}{dt} = M_t\, \mathbf{f}$, where $M_t = \text{diag}(\mathbf{x}_t) - \mathbf{x}_t \mathbf{x}_t^\top$. Approximating the derivative gives $\Delta \mathbf{x}_t \approx M_t \mathbf{f}$. We find the initial $\mathbf{f}$ by minimizing the sum of squared errors across all time intervals:

$$\min_{\mathbf{f}} \sum_t \|\Delta \mathbf{x}_t - M_t\, \mathbf{f}\|^2. \tag{S2}$$

This least-squares problem can be formulated as a quadratic program. Defining $Q = \sum_t M_t^\top M_t$ and $\mathbf{c} = \sum_t M_t^\top \Delta \mathbf{x}_t$, the objective becomes minimizing $\frac{1}{2}\mathbf{f}^\top (2Q)\, \mathbf{f} - (2\mathbf{c})^\top \mathbf{f}$. We add a small regularization term ϵI for numerical stability and impose the constraint $\sum_{i \in S} f_i = 0$ to set the mean fitness to zero, yielding the QP:

$$\min_{\mathbf{f}} \ \tfrac{1}{2}\mathbf{f}^\top (2Q + \epsilon I)\, \mathbf{f} - (2r)^\top \mathbf{f}, \quad \text{subject to} \sum_{i \in S} f_i = 0. \tag{S3}$$

Refinement via Joint Likelihood Optimization We refine the initial estimates $\{f_i\}$ by maximizing the likelihood of the observed counts $Y = \{y_{i,t}\}$ under the full replicator model. The analytical solution to Equation S1 for constant fitness is:

$$\hat{x}_i(t) = \frac{x_{0,i}\, \exp(f_i\, t)}{\sum_{j \in S} x_{0,j}\, \exp(f_j\, t)}, \tag{S4}$$

where $\{x_{0,i}\}$ are the initial frequencies at $t = 0$ ($\sum_{i \in S} x_{0,i} = 1$). Assuming the observed counts $Y_t = \{y_{i,t}\}$ at time t follow a multinomial distribution with N_t total trials (cells) and probabilities $\hat{\mathbf{x}}(t) = \{\hat{x}_i(t)\}$, the likelihood is:

$$P\big(Y_t \mid \hat{\mathbf{x}}(t), N_t\big) = \frac{N_t!}{\prod_{i \in S} y_{i,t}!} \prod_{i \in S} \hat{x}_i(t)^{\,y_{i,t}}. \tag{S5}$$

We maximize the total log-likelihood ($\sum_t \log P(Y_t \mid \hat{\mathbf{x}}(t), N_t)$) jointly over the fitness parameters $\{f_i\}$ and the initial frequencies $\{x_{0,i}\}$, subject to $\sum_{i \in S} f_i = 0$. The QP solution provides the starting values for the optimization.

bioRxiv preprint doi: https://doi.org/10.1101/2023.07.14.549079; this version posted June 21, 2025. The copyright holder for this preprint (which was not certified by peer review) is the author/funder, who has granted bioRxiv a license to display the preprint in perpetuity. It is made available under aCC-BY 4.0 International license.

Growth Offset Correction for Passaging Experiments In cell culture experiments involving passaging, the overall population growth rate can be estimated. If n_0 and n_b are the total cell counts at the start and end of a passage of duration Δt, the observed mean growth rate is $g_0 = \ln(n_b/n_0)/\Delta t$. We shift the inferred relative fitness values $\{f_i\}$ by a constant so that the mean fitness predicted by the replicator model ($\sum_i f_i \hat{x}_i(t)$, averaged over time) matches the observed mean growth rate g_0.

Bootstrapping To estimate uncertainty in fitness parameters, both the QP and maximum-likelihood fitting steps are repeated across multiple bootstrap resamples of the input count data $\{y_{it}\}$. The output of this step is a distribution of fitness estimates per each frequent karyotype.

1.3 Step 2: Fitness Estimation for one-MS-step neighbors

In this step, we extend fitness estimates to the sparsely observed karyotypes that are one gain or loss event ('one-MS-step neighbors') away from the frequent karyotypes identified in Step 1. Due to their low counts, full dynamic modeling is impractical. Instead, we approximate their fitness (f_i) based on the inferred mutational flux from their frequent parents ($j \in S$). This approach assumes that these neighbors primarily arise through such single missegregation events (governed by a missegregation probability $P(\alpha_i \mid \alpha_j)$ dependent on the per-chromosome rate p); that flux from rare-to-rare or rare-to-frequent types is negligible; and that the missegregation rate p is constant and known (or estimated). Let:

$$P(\alpha_i \mid \alpha_j) = \sum_{z=|\alpha_i-\alpha_j|}^{\alpha_j} \binom{\alpha_j}{z} p^z (1-p)^{\alpha_j - z} \, 0.5^z \binom{z}{\frac{z+\alpha_j-\alpha_i}{2}} \tag{S6}$$

be the probability of a missegregation event producing i (copy number α_i) from j (copy number α_j), where p is the per-chromosome missegregation rate [26, 19].

1.3.1 Fitness Inference via Flux Approximation and Likelihood

The rate of change of neighbor i's frequency $x_i(t)$ is approximated as:

$$\frac{dx_i}{dt} \approx \underbrace{\sum_{j \in S} P(\alpha_i \mid \alpha_j)\, f_j\, x_j(t)}_{\text{Flux from frequent parents}} + \underbrace{f_i\, x_i(t)}_{\text{Growth of neighbor } i}, \tag{S7}$$

neglecting missegregation from other rare types and back-mutation to frequent types. This ODE can be solved approximately. If we consider flux from a single parent j starting at effective time $t_{\text{birth},j}$ and approximate $x_j(t) \approx x_j(t_{\text{birth},j}) \exp(f_j\,[t - t_{\text{birth},j}])$, the contribution $x_i^j(t)$ from parent j evolves according to:

$$\frac{dx_i^j}{dt} = P(\alpha_i \mid \alpha_j)\, f_j\, x_j(t) + f_i\, x_i^j(t), \quad \text{with } x_i^j(t_{\text{birth},j}) = 0. \tag{S8}$$

The solution relates the ratio $x_i^j(t)/x_j(t)$ to the fitness difference $(f_i - f_j)$:

$$\frac{x_i^j(t)}{x_j(t)} = \frac{P(\alpha_i \mid \alpha_j)\, f_j}{f_i - f_j}\Big[\exp\big((f_i - f_j)\,[t - t_{\text{birth},j}]\big) - 1\Big]. \tag{S9}$$

The total expected frequency is $x_i(t) = \sum_{j \in S} x_i^j(t)$, under the approximation that x_i remains small.

We estimate f_i for neighbor i by maximizing a likelihood function that combines the probability of observing its counts $y_{i,t}$ with a prior on fitness differences relative to its parents. Let $\mathcal{P}(i) \subseteq S$ be the set of frequent parents of i. We assume fitness differences $\delta_{ij} = f_i - f_j$ follow a Gaussian distribution with

bioRxiv preprint doi: https://doi.org/10.1101/2023.07.14.549079; this version posted June 21, 2025. The copyright holder for this preprint (which was not certified by peer review) is the author/funder, who has granted bioRxiv a license to display the preprint in perpetuity. It is made available under aCC-BY 4.0 International license.

mean μ_δ and standard deviation σ_δ (estimated empirically from all neighbor-parent pairs). The likelihood for f_i is:

$$\mathcal{L}(f_i) = \underbrace{\prod_t \text{Binomial}\Big(y_{i,t} \,\Big|\, N_t^{\text{total}},\, x_i(t)\Big)}_{\text{Observation Likelihood}} \times \underbrace{\prod_{j \in \mathcal{P}(i)} \mathcal{N}\Big((f_i - f_j) \,\Big|\, \mu_\delta,\, \sigma_\delta^2\Big)}_{\text{Prior on Fitness Difference}}, \tag{S10}$$

where N_t^{total} is the total cell count at time t, and $x_i(t)$ depends on f_i via Equation S9. We maximize $\log \mathcal{L}(f_i)$ with respect to f_i.

The single-step neighbor fitnesses are estimated within the same boostrapping loop as the frequent karyotypes, using the same resampled data. Thus the output of this step is a distribution of fitness estimates per each neighbor karyotype.

1.4 Step 3: Fitness Inference for All Other Karyotypes via Kriging

To build a more comprehensive fitness landscape, this step estimates fitness for remaining viable karyotypes (those neither frequent nor direct one-MS-step neighbors of frequent types). We employ Gaussian process regression (Kriging) to interpolate fitness values across the karyotype space. This interpolation uses the fitness estimates derived in Steps 1 and 2 as anchor points. The core assumption underpinning this approach is that fitness constitutes a relatively smooth function over the space of karyotypes (represented by copy number vectors), and specifically that the chosen Matern kernel (with $\nu = 1.5$) adequately captures this smoothness structure. Karyotypes containing zero copies of any chromosome are deemed non-viable and excluded prior to interpolation. It is important to note that, as with any interpolation method, the reliability of Kriging predictions diminishes significantly for karyotypes distant from the anchor points in the high-dimensional space. Therefore, whilst in principle we could apply this interpolation across the entire karyotype space, in practice we limit our predictions to single-step neighbours of karyotypes with fitness estiamtes from the prior two steps. Kriging was implemented using the `Krig` function in the R package "`fields`", with all parameters except the aforementioned kernel set at default values. The default setting ($\nu = 1.5$) in `fields::Krig()` corresponds to an exponential kernel, which assumes the fitness landscape is maximally rough. This may understate the degree of local structure in karyotype space, where similar copy-number profiles often exhibit related fitness. Increasing ν to 1.5 allows for limited smoothness while preserving flexibility, in the absence of definitive information about the landscape's regularity.

1.4.1 Bootstrap Procedure for Uncertainty and Decorrelation

The Kriging step is performed within a separate bootstrap procedure. At every bootstrap iteration we keep the karyotype coordinates fixed but resample, with replacement, the anchor fitness estimates generated in Steps 1–2. A fresh Gaussian-process model is fitted to each resampled dataset and used to predict fitness over the full evaluation set of karyotypes. This helps in obtaining uncertainty estimates for the Kriging predictions and is specifically designed to reduce potential correlations between the errors in fitness estimates for frequent clones and the errors for their neighbors (which depend on the parent's fitness). By using a sufficient number of bootstrap iterations, we obtain a distribution of fitness predictions for each interpolated karyotype. We summarize this distribution by its mean and standard deviation, assuming normality, providing a compact representation of the inferred landscape and its uncertainty.

1.5 Assessing Internal Consistency: Cross-Validation Procedure

To evaluate the internal consistency and predictive reliability of the inferred fitness landscape, particularly in the absence of ground truth experimental data, we employ a cross-validation procedure. This procedure assesses the extent to which the fitness landscape exhibits local structure, specifically testing whether the fitness of any given karyotype and its immediate neighbors can be reasonably predicted from the

bioRxiv preprint doi: https://doi.org/10.1101/2023.07.14.549079; this version posted June 21, 2025. The copyright holder for this preprint (which was not certified by peer review) is the author/funder, who has granted bioRxiv a license to display the preprint in perpetuity. It is made available under aCC-BY 4.0 International license.

landscape inferred using data from other related karyotypes. A high degree of predictability indicates good generalization capability and robustness of the overall fitness landscape constructed in the preceding steps, while a low degree suggests otherwise.

1.5.1 Method

The procedure focuses on the frequent karyotypes and their neighbors: For each frequent karyotype $i \in S$:

1. Temporarily remove frequent karyotype i and all of its one-MS-step neighbors from the set of known fitness values (obtained in Sections 1.2 and 1.3).
2. Re-fit the Kriging model (Section 1.4) using the fitness data from the remaining frequent karyotypes and their neighbors.
3. Use this re-fitted Kriging model to predict the fitness values for the held-out karyotype i and its neighbors. Let these be the cross-validated predictions g_c.

This process is repeated for every frequent karyotype $i \in S$. Let $\mathcal{C}$ be the set of all karyotypes whose fitness was predicted during this CV procedure (i.e., all frequent karyotypes and all their one-MS-step neighbors). We compare the cross-validated predictions g_c for $c \in \mathcal{C}$ with the original estimates f_c obtained using the full dataset.

1.5.2 Cross-Validation Metric (R^2_X)

We quantify the agreement between the original estimates and the cross-validated predictions using a rescaled coefficient of determination, R^2_X:

$$R^2_X = 1 - \frac{\sum_{c \in \mathcal{C}} \left(f_c - g_c\right)^2}{\sum_{c \in \mathcal{C}} \left(f_c - \overline{f}\right)^2}, \quad \text{where } \overline{f} = \frac{1}{|\mathcal{C}|} \sum_{c \in \mathcal{C}} f_c. \tag{S11}$$

A high R^2_X suggests that the fitness estimates for individual karyotypes are well-supported by the surrounding fitness landscape inferred from other related clones, indicating internal consistency and good generalization. A low or negative R^2_X suggests potential issues like overfitting to noise in the frequency data of the held-out clone or poor interpolation by the Kriging model.

1.6 Predicting Future Evolution: Forecasting Simulation

ALFA-K can use the inferred fitness landscape (represented by mean fitness $\hat{f}_i$ and standard deviation σ_i for each karyotype i) to forecast future population dynamics. To predict dynamics of evolving populations, we used an agent-based model (Section 2).

1.6.1 Calculating the Steady-State Distribution

We determined the steady-state relative frequency distribution of co-existing karyotypes ($\mathbf{x}_{\text{ss}}$) under constant conditions by analyzing the linear system governing the dynamics of absolute cell abundances ($\mathbf{y}$) as described previously [26]. This system is described by the ordinary differential equation $\frac{d\mathbf{y}}{dt} = A'\mathbf{y}$, where the matrix A' incorporates both fitness-dependent growth and mutation rates derived from the per-division missegregation probabilities (Q). Specifically, the off-diagonal elements A'_{ij} ($i \neq j$) represent the rate at which type j produces type i via missegregation, given by the product of the parent fitness and the per-division probability ($A'_{ij} = f_j Q_{ij}$). The diagonal elements A'_{ii} represent the net growth

bioRxiv preprint doi: https://doi.org/10.1101/2023.07.14.549079; this version posted June 21, 2025. The copyright holder for this preprint (which was not certified by peer review) is the author/funder, who has granted bioRxiv a license to display the preprint in perpetuity. It is made available under aCC-BY 4.0 International license.

rate of type i, accounting for its intrinsic fitness f_i and its loss rate due to mutating into other types ($A'_{ii} = f_i(1 + Q_{ii})$, where $Q_{ii} = -\sum_{k \neq i} Q_{ki}$ is derived from the per-division probabilities). We numerically constructed this matrix A' using the inferred fitnesses $\mathbf{f}$ and the mutation probabilities Q derived from the per-chromosome rate p. The steady-state relative frequency distribution $\mathbf{x}_{\mathrm{ss}}$ corresponds to the normalized dominant eigenvector $\mathbf{v}_{max}$ of A'. This vector $\mathbf{v}_{max}$, associated with the eigenvalue of A' having the largest real part (which dictates the long-term asymptotic growth rate of the system), was computed efficiently using sparse matrix eigensolvers. Finally, the eigenvector was normalized such that its elements sum to one ($\mathbf{x}_{\mathrm{ss}} = \mathbf{v}_{max} / \sum_k v_{k,max}$) to yield the probability distribution representing the steady state.

==

2 Agent-Based Model (ABM)

2.1 Overview

We simulate the evolution of chromosome-level copy-number profiles in a population of cultured cells assumed to be well mixed, meaning that each cell experiences the same environment and competes equally with every other cell; no spatial structure is modelled. The simulator supports two use cases, corresponding to separate analyses in our manuscript:

Synthetic-data generation (GRF mode): Fitness is defined by a known Gaussian-random-field (GRF) function, allowing us to benchmark how accurately ALFA-K recovers the underlying landscape. **Forward prediction (LUT mode)**: Fitness values inferred by ALFA-K are supplied as a look-up table, and the simulator predicts future karyotype dynamics under those selective pressures.

Cells can only change in number or genotype through mitotic division. Division rates are karyotype specific; chromosomal missegregation during division generates variation. Spontaneous death is not explicitly modelled, but cells are removed if they produce inviable daughters (with zero copies of any chromosome class) or during periodic passaging (see 2.1).

Software implementation

The simulator is written in C++17 and exposed to R through `Rcpp`. Source code, documentation, and installation instructions are available in the `alfakR` package (`https://github.com/Richard-Beck/alfakR`).

Key model objects

- **Karyotypes** are stored as fixed-length integer vectors, one element per autosome. All copy numbers are strictly positive.
- **Population state** maps each unique karyotype to its current cell count.
- **Fitness specification** is supplied either as a numeric table (LUT mode) or by a Gaussian-random-field (GRF) function,

$$f(\alpha) = \frac{1}{\pi\sqrt{M}} \sum_{i=1}^{M} \sin\left(\frac{\|\alpha - \mathbf{r}_i\|_2}{\lambda}\right), \tag{S12}$$

  where α is the karyotype vector, $\{\mathbf{r}_i\}$ are user-defined centroids, and λ is the GRF wavelength parameter.

Stochastic division-and-segregation algorithm

At each discrete time increment Δt, the simulator processes every karyotype present (consistent with previous models [26, 19]) as follows:

bioRxiv preprint doi: https://doi.org/10.1101/2023.07.14.549079; this version posted June 21, 2025. The copyright holder for this preprint (which was not certified by peer review) is the author/funder, who has granted bioRxiv a license to display the preprint in perpetuity. It is made available under aCC-BY 4.0 International license.

1. **Number of divisions.** Let n_i be the number of cells with a given karyotype and f its fitness. The expected number of divisions is
$$\mu = n_i f \Delta t.$$
The realised number of divisions is sampled from a Poisson distribution with mean μ, but cannot exceed n_i.

2. **Segregation outcome.** Let C be the total number of chromosomes in the parent cell, and p the per-chromosome missegregation probability.

   - With probability $(1-p)^C$, the division is faithful and both daughters match the parent.
   - Otherwise, the division is error-prone. The number of chromosomes that mis-segregate is sampled as
   $$K \sim \text{Binomial}(C, p).$$

3. **Daughter formation.** Exactly K chromosomes are sampled uniformly without replacement from the C total. Each mis-segregating chromosome is randomly allocated to one daughter (with probability 1/2), resulting in a +1 copy number change in that daughter and −1 in the other. Any daughter with a zero in any chromosome class is discarded.

4. **Population update.** Parent cells that divided are removed; viable daughters are added to the population.

All stochastic sampling uses a Mersenne Twister engine, seeded by the user or from hardware entropy if no seed is provided.

Population control (serial passaging)

To emulate the dynamics of cell-culture and xenograft experiments—where cells grow to capacity and are then re-seeded at lower density—the simulator imposes a user-defined maximum population size $N_{\max}$. When the total cell count exceeds this threshold, each karyotype is independently down-sampled via a binomial draw that retains a fixed fraction s of its cells. This reduces the total number of cells while preserving relative karyotype abundances.

3 Validation of ALFA-K using Synthetic Data

To rigorously evaluate ALFA-K's performance and understand its limitations, we generated synthetic datasets where the ground truth fitness landscape and evolutionary dynamics are known. This approach allows us to directly compare inferred fitness landscapes against the known ground truth, assess the impact of factors such as landscape complexity, sampling frequency, and noise on inference accuracy, evaluate the reliability of the cross-validation score (R^2_X) as a diagnostic tool, and test the accuracy of evolutionary forecasts against known future population states. These synthetic datasets were generated using an Agent-Based Model (ABM) to simulate population evolution on Gaussian Random Field (GRF) fitness landscapes.

3.0.1 Simulation Setup and Parameters

We ran ABM simulations for 300 days on GRF landscapes with varying complexity (λ). Key parameters are listed in Table S1. Populations evolving on complex landscapes (low λ) often exhibited punctuated evolutionary dynamics, whereas those on smoother landscapes (high λ) showed more gradual fitness increases (Fig. S1A-D).

bioRxiv preprint doi: https://doi.org/10.1101/2023.07.14.549079; this version posted June 21, 2025. The copyright holder for this preprint (which was not certified by peer review) is the author/funder, who has granted bioRxiv a license to display the preprint in perpetuity. It is made available under aCC-BY 4.0 International license.

Description	Symbol	Sim 1	Sim 2	Sim 3	Comments
Per-chromosome missegregation probability	p		5×10^{-5}		$(10^{-5}$–$10^{-1})$ [2, 35, 36]
Time increment per step	Δt		0.1		–
Number of simulation steps	ℓ	variable	3000	variable	–
Population cap (passaging threshold)	$n_{\max}$	10^7	2×10^6	2×10^6	Corresponds to small tumour fragments or large cultures [37]
Survival fraction after passaging	s		0.01		Typical value for seeding PDX models [38]
Initial population size	N_0	10^5	5×10^4	2×10^5	Typical values for seeding PDX models [39]
GRF wavelength	λ	–	0.2–1.6	–	
GRF centroids	$\{\mathbf{r}_i\}$	y	n	y	–
Fitness table (LUT mode only)	—	y	n	y	–

Table S1: Parameters used in ABM simulations. ABM simulations are used: to predict karyotype evolution for empirical datasets (Sim 1); to generate synthetic data to test ALFA-K (Sim 2); and to predict evolution of the synthetic cell populations based on ALFA-K fitness landscapes (Sim 3). $\{\mathbf{r}_i\}$ and the fitness table represent optional arguments whose respective presence or absence is indicated with 'y' or 'n'.

For testing ALFA-K, we sampled data (simulating experimental measurements) from these simulations. We extracted longitudinal count data for 2, 4, or 8 consecutive passages ending around day 120 of the simulation (Fig. S1E). This timeframe usually captured populations during active adaptation, allowing us to test ALFA-K's ability to infer ongoing dynamics and predict future evolution.

3.1 Metrics for Performance Evaluation

We used several metrics to compare ALFA-K's inferred fitness landscapes (f^{pred}) against the ground truth (f^{true}) from the GRF, and to compare forecasted population states ($\mathbf{x}^p$) against the true future states from the ABM ($\mathbf{x}^a$).

Variables for Metrics Let f^{pred} and f^{true} be predicted and true fitness vectors. Let $\mathbf{x}^p$, $\mathbf{x}^a$, and $\mathbf{x}^0$ be predicted, actual, and baseline frequency vectors, respectively. Let $\mathbf{k}_i$ be the vector representation of karyotype i. The centroid is $c(\mathbf{x}) = \sum_i \mathbf{k}_i x_i / \sum_i x_i$.

Spearman's Correlation (ρ) Measures rank correlation between f^{pred} and f^{true}:

$$\rho = \mathrm{corr}\Big(\mathrm{rank}(f^{pred}), \mathrm{rank}(f^{true})\Big). \tag{S13}$$

bioRxiv preprint doi: https://doi.org/10.1101/2023.07.14.549079; this version posted June 21, 2025. The copyright holder for this preprint (which was not certified by peer review) is the author/funder, who has granted bioRxiv a license to display the preprint in perpetuity. It is made available under aCC-BY 4.0 International license.

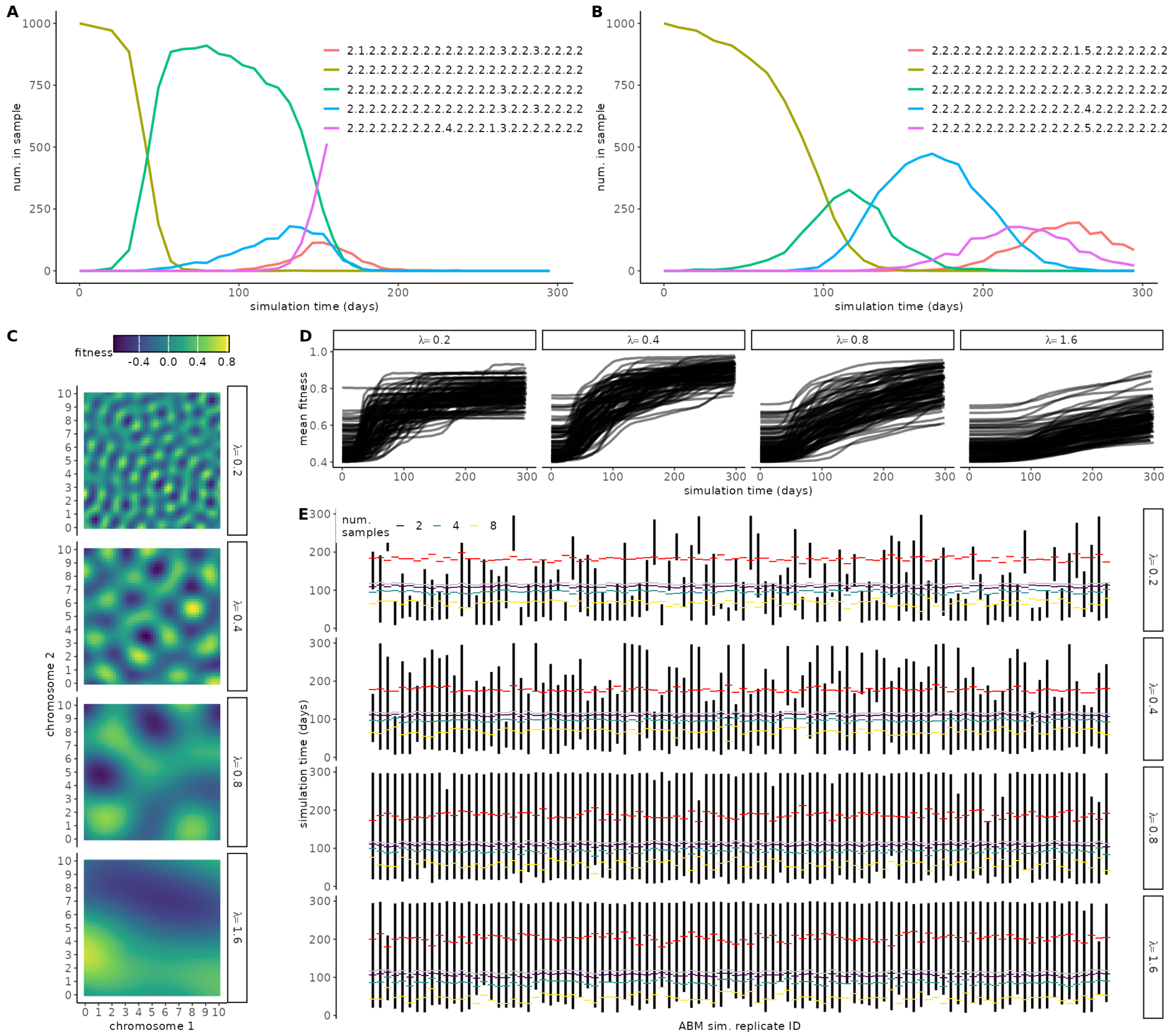

Figure S1: In silico test populations and sampling strategy. (A-B) Example simulation output for ABM cell populations evolving on GRF fitness landscape with A) $\lambda = 0.2$ or B) $\lambda = 1.6$. Each coloured line represents the longitudinal frequency of a different karyotype. (C) Increasing the wavelength (λ) results in GRF with decreasing complexity. (D) Mean fitness of ABM cell populations evolving on artificial fitness landscapes of varying complexity (as determined by λ). (E) Overview of ABM sampling strategy. Black bars represent the longest period of continuous evolutionary progress in each ABM simulation as measured by consecutive passages with fitness increases. Colored points indicate the time intervals included in ALFA-K training for different numbers of timepoints, with grey points representing the latest included timepoints and red points showing the latest predictions.

Pearson's Correlation (r) Measures linear correlation between f^{pred} and f^{true}:

$$r = \frac{\sum_i (f_i^{pred} - \overline{f^{pred}})(f_i^{true} - \overline{f^{true}})}{\sqrt{\sum_i (f_i^{pred} - \overline{f^{pred}})^2}\sqrt{\sum_i (f_i^{true} - \overline{f^{true}})^2}}. \quad \text{(S14)}$$

bioRxiv preprint doi: https://doi.org/10.1101/2023.07.14.549079; this version posted June 21, 2025. The copyright holder for this preprint (which was not certified by peer review) is the author/funder, who has granted bioRxiv a license to display the preprint in perpetuity. It is made available under aCC-BY 4.0 International license.

Rescaled R^2 (R^2, R^2_F, R^2_X) Proportion of variance explained, computed on mean-centered data:

$$R^2 = 1 - \frac{\sum_i[(f_i^{true} - \overline{f^{true}}) - (f_i^{pred} - \overline{f^{pred}})]^2}{\sum_i(f_i^{true} - \overline{f^{true}})^2}. \tag{S15}$$

R^2_F is computed on frequent karyotypes only. R^2_X is the cross-validation metric (Eq. S11).

Angle Metric (θ) Quantifies directional alignment of trajectories via centroids. Displacement vectors: $\mathbf{v}^p = c(\mathbf{x}^p) - c(\mathbf{x}^0)$, $\mathbf{v}^a = c(\mathbf{x}^a) - c(\mathbf{x}^0)$.

$$\theta = \arccos\left(\frac{\mathbf{v}^p \cdot \mathbf{v}^a}{\|\mathbf{v}^p\|\|\mathbf{v}^a\|}\right). \quad \text{(Smaller } \theta \text{ is better)} \tag{S16}$$

The null distribution CDF for θ between two random unit vectors in $\mathbb{R}^N$ is:

$$F(\theta) = \int_0^\theta \sin^{N-2}(t)\,dt \Big/ \int_0^\pi \sin^{N-2}(t)\,dt. \tag{S17}$$

Cosine Similarity (CS) Compares frequency profiles $\mathbf{x}^p$ and $\mathbf{x}^a$:

$$\text{CS}(\mathbf{x}^p, \mathbf{x}^a) = \frac{\sum_i x_i^p\, x_i^a}{\sqrt{\sum_i (x_i^p)^2}\,\sqrt{\sum_i (x_i^a)^2}}. \quad \text{(Closer to 1 is better)} \tag{S18}$$

Euclidean Distance (d_E) Distance between centroids $c(\mathbf{x}^p)$ and $c(\mathbf{x}^a)$:

$$d_E = \|\,c(\mathbf{x}^p) - c(\mathbf{x}^a)\|. \quad \text{(Smaller is better)} \tag{S19}$$

Overlap Coefficient (Ω) Fraction of shared population mass:

$$\Omega(\mathbf{x}^p, \mathbf{x}^a) = \frac{\sum_i \min(x_i^p, x_i^a)}{\min\left(\sum_i x_i^p,\ \sum_i x_i^a\right)}. \quad \text{(Closer to 1 is better)} \tag{S20}$$

Wasserstein Distance (d_W) Earth Mover's Distance between distributions $\mathbf{x}^p$ and $\mathbf{x}^a$, requiring minimal cost to transform one to the other based on euclidean distance $d(\mathbf{k}_1, \mathbf{k}_2)$ between karyotypes:

$$d_W(\mathbf{x}^p, \mathbf{x}^a) = \min_{\gamma \in \Pi(\mathbf{x}^p, \mathbf{x}^a)} \sum_{i,j} \left\|\mathbf{k}_i - \mathbf{k}_j\right\|_2 \gamma_{ij}, \tag{S21}$$

where $\Pi(\mathbf{x}^p, \mathbf{x}^a)$ is the set of transport plans whose row and column sums equal $\mathbf{x}^p$ and $\mathbf{x}^a$, respectively. A smaller d_W indicates that the predicted distribution is closer to the actual one.

3.2 Evaluating Fitness Landscape Inference Accuracy

We applied ALFA-K to the sampled synthetic data and compared the inferred fitness landscapes to the known GRF ground truth using metrics defined in Section 3.1. Accuracy generally improved with smoother landscapes (higher λ), a higher threshold N for defining frequent karyotypes, and more sampled time points (Fig. S2A). These factors help mitigate the impact of sampling noise and demographic stochasticity, especially for low-frequency clones.

The analysis revealed that accurate fitness estimation for the initial set of frequent karyotypes is crucial. Fits where the frequent-subset accuracy (R^2_F) was poor, or where the frequent set was very small, rarely yielded accurate global landscapes (positive global R^2) (Fig. S2B). Errors in the initial frequent set estimation tend to propagate through the neighbor extension and Kriging steps. An example simulation

bioRxiv preprint doi: https://doi.org/10.1101/2023.07.14.549079; this version posted June 21, 2025. The copyright holder for this preprint (which was not certified by peer review) is the author/funder, who has granted bioRxiv a license to display the preprint in perpetuity. It is made available under aCC-BY 4.0 International license.

highlights this (Fig. S2C-E). With limited training data (2 passages), stochastic fluctuations in frequency trajectories could lead to incorrect ranking of closely competing clones. Providing more data (8 passages) allowed ALFA-K to resolve the long-term trends and correctly infer the fitness ranking, even with the same N threshold.

A Sankey diagram summarizing all fits (Fig. S2F) confirmed that poor global accuracy ($R^2 \leq 0$) was strongly associated with either poor initial frequent set accuracy ($R^2_F \leq 0$) or an insufficient number of frequent karyotypes identified, regardless of landscape complexity or number of passages. This underscores the importance of the initial frequent karyotype identification and fitness estimation step.

3.3 Evaluating Cross-Validation as a Diagnostic Heuristic

Since ground truth fitness is unknown in real experiments, we evaluated the utility of the cross-validation score R^2_X (Section 1.5) as a proxy for inference reliability using our synthetic data. The R^2_X scores calculated on the synthetic datasets showed trends consistent with direct accuracy metrics: scores were lower for more rugged landscapes (low λ) and when fewer training passages were used (Fig. S3A). This suggests R^2_X captures aspects related to the difficulty of inference. Importantly, we found a strong correlation between the sign of the cross-validation score and the sign of the true accuracy (R^2) against the ground truth. Fits achieving a positive R^2_X were significantly enriched for also having a positive R^2 (Fig. S3B). Furthermore, landscapes inferred with $R^2_X > 0$ showed substantially higher accuracy across all metrics (ρ, r, R^2) compared to those with $R^2_X \leq 0$ (Fig. S3C). These results support the use of R^2_X as a diagnostic heuristic. While not a guarantee of accuracy, a positive R^2_X indicates internal consistency and suggests the inferred landscape is more likely to be reliable.

3.4 Evaluating Evolutionary Forecasting Performance

Finally, we tested the ability of ALFA-K's inferred fitness landscapes to predict future evolutionary dynamics in the ABM simulations. We used the forecasting method described in Section 1.6 to simulate evolution forward from the last training time point and compared the predicted population state to the true state from the ABM simulation at later times.

3.4.1 Directional Accuracy (Angle Metric)

We assessed directional accuracy using the angle metric θ (Section 3.1), where lower values indicate better alignment between predicted and true evolutionary vectors. ALFA-K forecasts consistently achieved better-than-random directional accuracy (ECDFs shifted left from the null distribution, Fig. S4A). Accuracy generally improved with more training samples and decreased slightly for longer prediction horizons (more passages forecast). Crucially, forecasts generated from landscapes with good internal consistency ($R^2_X > 0$) were significantly more accurate directionally than those from landscapes with $R^2_X \leq 0$.

3.4.2 Comparison Against No-Evolution Baselines

We also compared ALFA-K forecasts against simple baseline models that assume no evolution (i.e., predicting the final observed state persists). We measured the fraction of times ALFA-K forecasts were 'better' than the baseline according to various metrics (Cosine Similarity, Euclidean Distance, etc.). When trained on sufficient data (e.g., 4+ passages) and achieving a positive cross-validation score ($R^2_X > 0$), ALFA-K forecasts outperformed these static baselines in roughly 75% of cases across different metrics and forecast horizons (Fig. S4B). In contrast, forecasts from fits with $R^2_X \leq 0$ rarely surpassed the baselines.

bioRxiv preprint doi: https://doi.org/10.1101/2023.07.14.549079; this version posted June 21, 2025. The copyright holder for this preprint (which was not certified by peer review) is the author/funder, who has granted bioRxiv a license to display the preprint in perpetuity. It is made available under aCC-BY 4.0 International license.

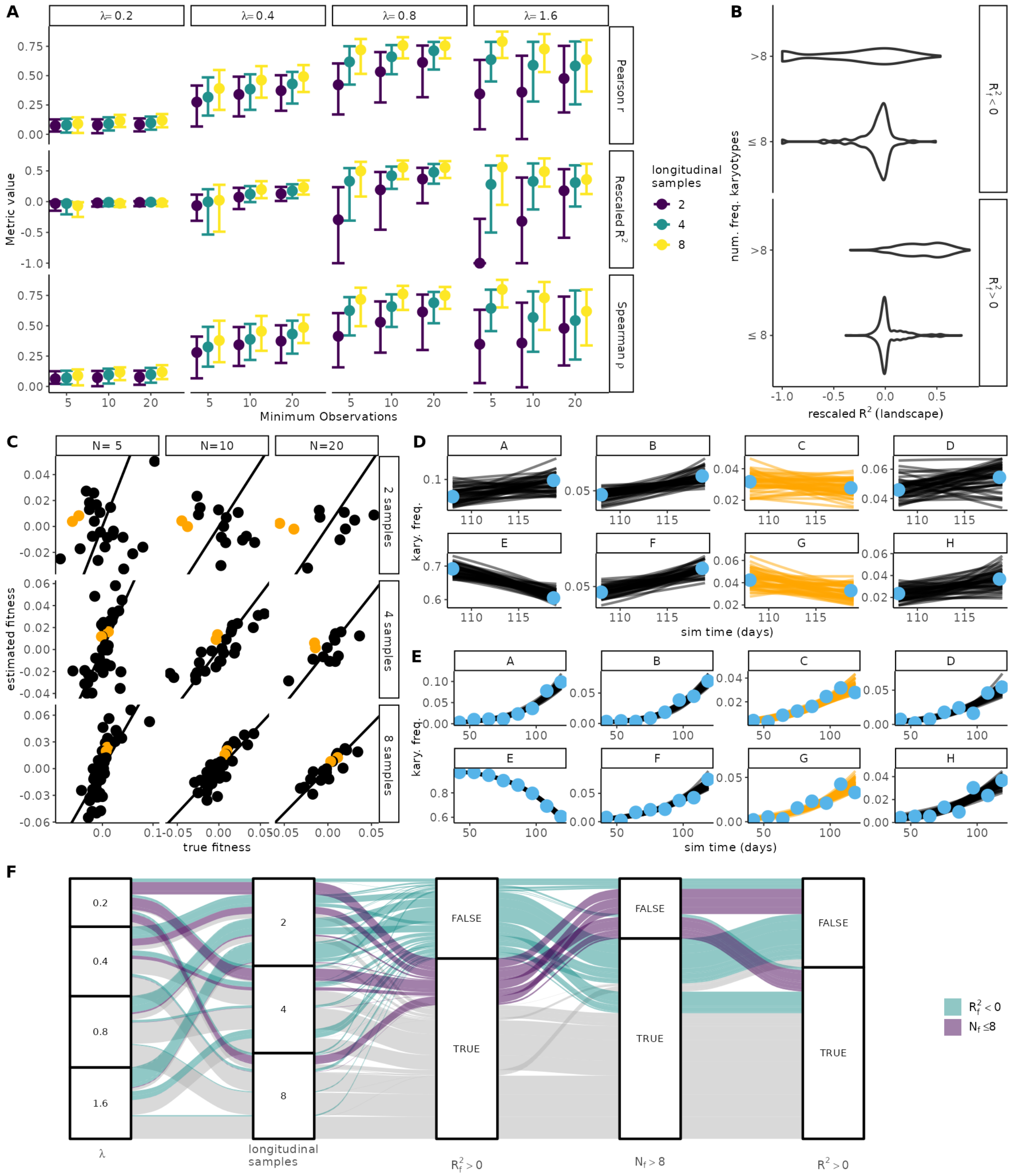

Figure S2: Performance of ALFA-K fitness inference on simulated landscapes. (A) Accuracy metrics (ρ, r, R^2; rows) vs. ground truth GRF fitness, shown as a function of landscape complexity (λ, columns), frequent karyotype threshold (N, x-axis), and number of sampled time points (colour). Boxes: 10th, 50th, 90th percentiles. (B) Global rescaled R^2 vs. frequent-subset R^2_F and number of frequent karyotypes identified. (C) Example: Estimated vs. true fitness for one simulation ($\lambda = 0.8$) with varying hyperparameters. Orange points highlight three clones whose rank order is inverted with limited data. (D–E) Frequency trajectories (observed points, fitted lines) for the top 8 karyotypes from (C) using $N = 20$ and (D) 2 passages or (E) 8 passages. Orange lines correspond to the mis-ranked clones in (C, D). (F) Sankey diagram summarizing accuracy across all fits, stratified by input parameters and intermediate results (sign of R^2_F, number of frequent karyotypes abbreviated as N_F) leading to final global R^2 sign.

3.4.3 Conclusion from Forecasting Evaluation

The forecasting results further validate ALFA-K and the utility of the R^2_X diagnostic. Landscapes deemed reliable by cross-validation not only reflect the known fitness landscape more accurately but also possess

bioRxiv preprint doi: https://doi.org/10.1101/2023.07.14.549079; this version posted June 21, 2025. The copyright holder for this preprint (which was not certified by peer review) is the author/funder, who has granted bioRxiv a license to display the preprint in perpetuity. It is made available under aCC-BY 4.0 International license.

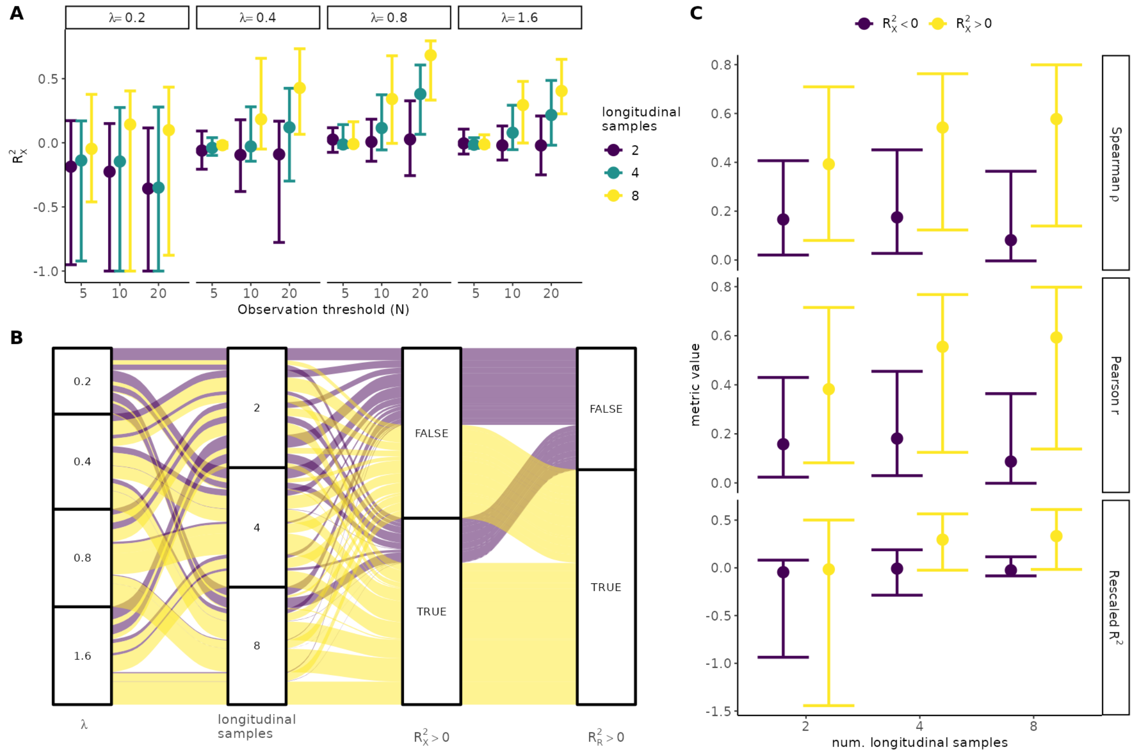

Figure S3: Cross-validation metric (R^2_X) as a diagnostic heuristic for ALFA-K performance on ABM data. (A) Cross-validation scores (R^2_X) vs. landscape complexity (λ, columns), frequent karyotype threshold (N, x-axis), and number of sampled time points (colour). Boxes: 10th, 50th, 90th percentiles. (B) Sankey diagram showing how filtering fits based on the sign of R^2_X enriches for fits with positive accuracy against ground truth ($R^2 > 0$). (C) Comparison of accuracy metrics (ρ, r, R^2) against ground truth, stratified by the sign of the cross-validation score ($R^2_X > 0$ vs. $R^2_X \leq 0$). Boxes: 10th, 50th, 90th percentiles.

significantly better predictive power regarding future evolutionary trajectories compared to unreliable landscapes or simple no-evolution assumptions.

4 Application to Experimental Data

ALFA-K was also applied to real-world longitudinal single-cell DNA sequencing data to infer fitness landscapes and forecast evolution in experimental systems, as described in the main text. Here we detail the source and processing of this data.

4.1 Data Source and Experimental Systems

The experimental data analyzed in this study originates from the longitudinal single-cell copy number sequencing dataset published by Salehi et al. [23]. Their work characterized karyotype evolution in two experimental systems: immortalized human mammary epithelial cell lines (184-hTERT, including wild-type *TP53*^{WT} and two independent *TP53*$^{\text{-/-}}$ lines) serially passaged for multiple generations, and four PDX models serially passaged in mice. Salehi and colleagues generated per-cell integer copy-number profiles across fixed genomic bins with the HMMCopy pipeline and applied the quality-control filters detailed in their publication [23].

Post-QC data were provided as a cell by genomic-bin integer matrix. A small number of samples were omitted because their reported genomic loci were inconsistent with the reference coordinates. Only bins from chromosomes 1–22 were retained. For each cell, the modal copy number across all bins assigned

bioRxiv preprint doi: https://doi.org/10.1101/2023.07.14.549079; this version posted June 21, 2025. The copyright holder for this preprint (which was not certified by peer review) is the author/funder, who has granted bioRxiv a license to display the preprint in perpetuity. It is made available under aCC-BY 4.0 International license.

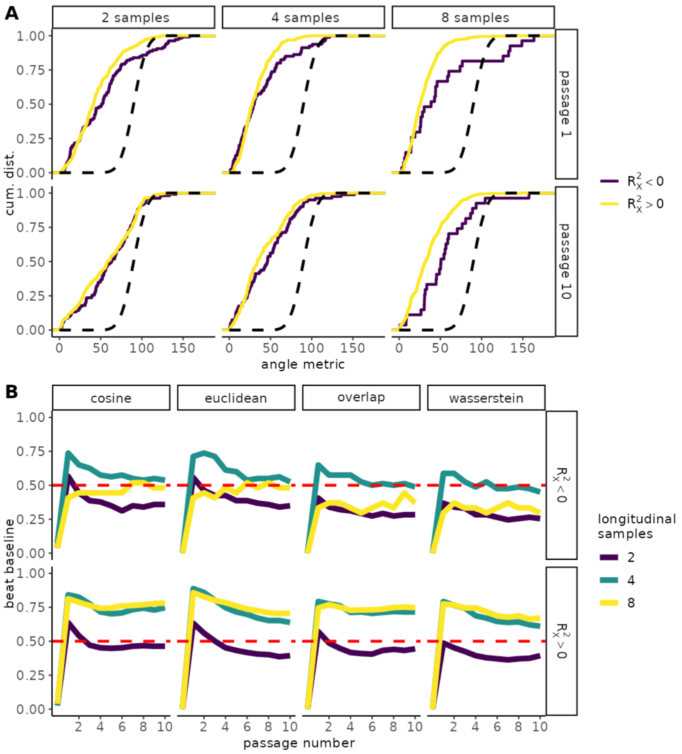

Figure S4: Evaluating the forecasting performance of ALFA-K fitness landscapes using ABM simulations. (**A**) Directional accuracy assessed by the angle metric θ. Empirical cumulative distribution functions (ECDFs) of θ are shown, faceted by number of training samples (columns) and forecast horizon (rows, in passages). Lines show the theoretical null distribution (black dashed) and results for ALFA-K fits stratified by cross-validation score ($R^2_X > 0$ vs. $R^2_X \leq 0$, colored lines). Left-shifted ECDFs indicate better directional accuracy. (**B**) Performance relative to no-evolution baselines. Facets show the fraction of ALFA-K forecasts outperforming a given baseline metric (columns; see Section 3.1) stratified by cross-validation score (rows). X-axis is the forecast horizon (passages); colored lines indicate number of training samples. Higher fractions indicate better relative performance for ALFA-K.

to the same chromosome was taken as that chromosome's copy number, yielding a 22-element integer karyotype vector; sex chromosomes were ignored. Experimental populations in the Salehi et al. dataset were serially passaged and sampled at multiple time points. Within a population, we defined the chronologically succeeding sample as the daughter of the immediately preceding sample, and every chain of at least two successive samples was kept as a lineage. For every lineage, chromosome-level karyotypes were grouped by sampling passage, and the number of cells displaying each distinct karyotype was tallied. These karyotype-by-passage matrices, paired with an assumed passage interval of 15 days (PDX) or 5 days (184-hTERT), constituted the inputs for ALFA-K fitness-inference analyses.

4.2 Modeling the Influence of Karyotype Background on Fitness Effects

Data Processing and Trajectory Selection. Longitudinal copy–number data were expressed as a series of transitions, each defined by two consecutive population samples. Every ALFA-K fit corresponds to a trajectory—an ordered list of these transitions—along with its length (n_{trans}). Within each bootstrap iteration trajectories were selected at random with probability proportional to (n_{trans}) and retained only if no transitions had appeared in a trajectory already chosen. This was repeated until no more non-overlapping trajectories remained. This procedure maximises coverage of long evolutionary paths while ensuring that each transition is analysed exactly once. Trajectories containing a single transition and those with negative CV scores were excluded.

bioRxiv preprint doi: https://doi.org/10.1101/2023.07.14.549079; this version posted June 21, 2025. The copyright holder for this preprint (which was not certified by peer review) is the author/funder, who has granted bioRxiv a license to display the preprint in perpetuity. It is made available under aCC-BY 4.0 International license.

Modeling Fitness Effect Distributions (GLMMs) Generalized Linear Mixed Models (GLMMs) were employed to analyze how context and treatment affect the properties of the Δf distributions, specifically their magnitude and variance, while accounting for the nested structure of the data using the `glmmTMB` R package.

Model for Absolute Fitness Effect Magnitude ($|\Delta f|$): Let R_{ijk} be the scaled absolute fitness effect ($|\Delta f| + 10^{-6}$) for the k-th potential mutation relative to the j-th focal karyotype within the i-th trajectory fit. The model treats R_{ijk} as Gamma distributed with mean μ_{ijk} and dispersion ϕ:

$$R_{ijk} \sim \text{Gamma}(\text{mean} = \mu_{ijk}, \text{dispersion} = \phi) \tag{S22}$$

The relationship between the mean and the fixed and random effects is modeled via a log link:

$$\log(\mu_{ijk}) = \beta_0 + \beta_{\text{context}} Z_{\text{context},i} + \beta_{\text{treat}} Z_{\text{treat},i} + u_{pdx[i]} + u_{fi[i]} + u_{k\_id[ij]}. \tag{S23}$$

Where:

- β_0 is the overall intercept.
- β_{context} and β_{treat} are the fixed effects coefficients for context (PDX vs. in vitro) and treatment (cisplatin vs. control, with cisplatin as the baseline level). Z denotes the corresponding indicator variables.
- $u_{pdx[i]} \sim \mathcal{N}(0, \sigma^2_{pdx})$ is the random intercept for the PDX/cell line associated with trajectory i.
- $u_{fi[i]} \sim \mathcal{N}(0, \sigma^2_{fi})$ is the random intercept for trajectory i, nested within PDX line.
- $u_{k\_id[ij]} \sim \mathcal{N}(0, \sigma^2_{k\_id})$ is the random intercept for the j-th focal karyotype, nested within trajectory i.

This model structure was fitted separately testing 'context' and 'treat' predictors (for 'treat', only PDX data was used).

Model for Variance of Fitness Effects ($\log(\textbf{Var}(\Delta f))$): Let V_{ij} be the variance of the Δf values across all potential mutations relative to the j-th focal karyotype within the i-th trajectory fit. The response variable is $U_{ij} = \log(V_{ij} + 10^{-6})$, scaled. This model treats U_{ij} as normally distributed:

$$U_{ij} \sim \mathcal{N}(\mu_{ij}, \sigma^2_{res}). \tag{S24}$$

The mean μ_{ij} is modeled linearly:

$$\mu_{ij} = \beta_0 + \beta_{\text{context}} Z_{\text{context},i} + \beta_{\text{treat}} Z_{\text{treat},i} + u_{pdx[i]} + u_{fi[i]}. \tag{S25}$$

Where terms are defined similarly to the prior model (Eq. S23), but random effects ($u_{pdx[i]} \sim \mathcal{N}(0, \sigma^2_{pdx})$, $u_{fi[i]} \sim \mathcal{N}(0, \sigma^2_{fi})$) are only needed up to the trajectory level as each data point U_{ij} represents one karyotype. σ^2_{res} is the residual variance.

Estimating Fitness-Landscape Correlation Length (LMM) Following the definition of correlation length in fitness landscapes formulated by [40], a long correlation length indicates a smooth landscape in which many single-chromosome changes are required to randomize fitness, while short correlation length characterizes a highly rugged landscape. To estimate this quantity we performed pairwise comparisons between the Δf vectors (v_p, v_q) of different karyotypes (p, q). The Pearson correlation ($sim_{pq} = \text{cor}(v_p, v_q)$) and Manhattan distance (dk_{pq}) were calculated. A Linear Mixed Model (LMM) was used to assess how similarity decays with distance using the `lme4` R package. The correlation sim_{pq} was transformed using

bioRxiv preprint doi: https://doi.org/10.1101/2023.07.14.549079; this version posted June 21, 2025. The copyright holder for this preprint (which was not certified by peer review) is the author/funder, who has granted bioRxiv a license to display the preprint in perpetuity. It is made available under aCC-BY 4.0 International license.

the Fisher-z transformation ($G_{pq} = \text{atanh}(sim_{pq})$) to stabilize variance and approximate normality. The distance dk_{pq} was transformed using $\log(1 + dk_{pq})$ to potentially linearize the relationship and handle $dk = 0$. The model structure was:

$$G_{pq} \sim \mathcal{N}(\mu_{pq}, \sigma^2_{res}), \tag{S26}$$

with:

$$\mu_{pq} = \beta_{\text{fixed}} + u_{fi1[p]} + u_{fi2[q]}. \tag{S27}$$

Where β_{fixed} represents the combined fixed effects part including main effects for $\log(1+dk_{pq})$, pair type (same trajectory, parallel, different line), treatment pairing, and interaction terms between distance and pair type. Random intercepts $u_{fi1[p]} \sim \mathcal{N}(0, \sigma^2_{fi1})$ and $u_{fi2[q]} \sim \mathcal{N}(0, \sigma^2_{fi2})$ accounted for non-independence arising from karyotypes belonging to specific trajectories.

Trajectory Dynamics The overlap in clonal composition between successive passages (sum of minimum frequencies) and the angular similarity between vectors of karyotypic change across passages were calculated to assess the rate and directionality of evolution.

Modeling of Whole-Genome Doubling (WGD) Effects

Karyotypes were classified as WGD$^+$ if their modal chromosome copy number was ≥ 3, and WGD$^-$ otherwise. Aneuploidy level for a karyotype $\mathbf{k}$ was quantified as the number of altered chromosomes, $d(\mathbf{k}) = \sum_i \mathbb{I}(k_i \neq \text{mode}(\mathbf{k}))$, where k_i is the copy number of chromosome i.

Aneuploidy Divergence The dynamics of aneuploidy accumulation over passages were modeled using non-linear least squares (via R's `nls` function), fitting the average number of altered chromosomes per cell (n_a) at passage t to an exponential saturation curve, $n_a = A(1 - e^{-\kappa t})$. Both the asymptote parameter (A) and the rate constant parameter (κ) were modeled using additive fixed effects for WGD status ($s \in \{\text{WGD}^-, \text{WGD}^+\}$) and experimental trajectory ($j \in \{\text{TrajA}, \text{TrajB}\}$), assuming no interaction: $A_{sj} = \beta_{A0} + \beta_{A,WGD} \times \mathbb{I}(s = \text{WGD}^+) + \beta_{A,Traj} \times \mathbb{I}(j = \text{TrajB})$ and $\kappa_{sj} = \beta_{\kappa 0} + \beta_{\kappa,WGD} \times \mathbb{I}(s = \text{WGD}^+) + \beta_{\kappa,Traj} \times \mathbb{I}(j = \text{TrajB})$, where $\mathbb{I}(\cdot)$ is the indicator function.

Fitness Effect Distributions Distributions of single-chromosome gain/loss fitness effects (Δf) for WGD$^+$ vs WGD$^-$ states were compared using a permutation Kolmogorov-Smirnov test. Mean effects were calculated per WGD status within each landscape fit before comparison. Significance was assessed against 10^4 permutations of WGD labels among fits ($P < 0.0001$).
